# A Critical Function of Mad2l2 in Primordial Germ Cell Development of Mice

**DOI:** 10.1371/journal.pgen.1003712

**Published:** 2013-08-29

**Authors:** Mehdi Pirouz, Sven Pilarski, Michael Kessel

**Affiliations:** Department of Molecular Cell Biology, Max Planck Institute for Biophysical Chemistry, Göttingen, Germany; Stanford University School of Medicine, United States of America

## Abstract

The development of primordial germ cells (PGCs) involves several waves of epigenetic reprogramming. A major step is following specification and involves the transition from the stably suppressive histone modification H3K9me2 to the more flexible, still repressive H3K27me3, while PGCs are arrested in G2 phase of their cycle. The significance and underlying molecular mechanism of this transition were so far unknown. Here, we generated mutant mice for the Mad2l2 (Mad2B, Rev7) gene product, and found that they are infertile in both males and females. We demonstrated that Mad2l2 is essential for PGC, but not somatic development. PGCs were specified normally in Mad2l2^−/−^ embryos, but became eliminated by apoptosis during the subsequent phase of epigenetic reprogramming. A majority of knockout PGCs failed to arrest in the G2 phase, and did not switch from a H3K9me2 to a H3K27me3 configuration. By the analysis of transfected fibroblasts we found that the interaction of Mad2l2 with the histone methyltransferases G9a and GLP lead to a downregulation of H3K9me2. The inhibitory binding of Mad2l2 to Cyclin dependent kinase 1 (Cdk1) could arrest the cell cycle in the G2 phase, and also allowed another histone methyltransferase, Ezh2, to upregulate H3K27me3. Together, these results demonstrate the potential of Mad2l2 in the regulation of both cell cycle and the epigenetic status. The function of Mad2l2 is essential in PGCs, and thus of high relevance for fertility.

## Author Summary

Primordial germ cells (PGCs) are the origin of sperm and oocytes, and are responsible for transferring genetic information to the next generation faithfully. PGCs are first specified from pluripotent epiblast cells early in embryonic development. Second, they reprogram their epigenetic signature by changing histone modifications. This developmental event is specific to germ cells but not somatic cells. Although many players in the specification of PGCs are identified, only little is known about the genes essential for the regulation of the second phase. Here, we report that the Mad2l2 gene product plays an important role in the epigenetic reprogramming of PGCs. In wild type PGCs the cell cycle is arrested, and the methylation of histone 3 on residue K9 is replaced by methylation on K27. Our findings indicate that Mad2l2 is involved in this coordination of cell cycle and epigenetic reprogramming. The elucidation of this mechanism would help to identify the genetic basis of infertility.

## Introduction

In mice, PGCs are induced by BMP signaling at the onset of gastrulation at day 7.25 of embryonic development (E7.25) in the posterior epiblast. They enter the extraembryonic mesoderm and the hindgut endoderm, and then migrate through the dorsal mesentery, until they accumulate in the genital ridges to participate in the generation of the future gonads [1]. Once specified, PGCs undergo various changes of their transcriptional profile and epigenetic status, which together establish the unique germ cell fate separate from surrounding somatic cells [2], [3]. Two PR-domain containing proteins, Prdm1 (Blimp1) and Prdm14, initiate the PGC-specific program [4], [5]. The reactivation of the pluripotency-associated gene Sox2 that had been silenced in the epiblast of the egg cylinder is an immediate early change upon PGC specification [6], [7]. It leads to the acquisition of a potential to become pluripotent under specific culture conditions [8]–[10]. Around E7.5 the transcription of somatic genes like Hox, Snail or Brachyury become repressed as a result of Prdm1 function, and the characteristic PGC gene Dppa3 becomes upregulated. Together, the typical transcriptional signature of PGCs has developed by E9.0 [11].

The chromatin of PGCs undergoes extensive remodeling, affecting both DNA and histone configurations [3], [12]. De novo DNA methylation is suppressed as the result of the downregulation of the DNA methyltransferases Dnmt3b and Uhrf1 [7]. Consequently, a passive DNA demethylation is initiated at around E8.0, and by E9.5, PGCs become hypomethylated [3]. At E7.75, PGCs harbor a high, genome-wide level of the repressive histone modification H3K9me2, similar to the surrounding somatic cells. This modification is gradually lost, and by E9.25 suppressed in most PGCs. The corresponding histone methyltransferases GLP and G9a, which methylate lysine residue 9 of histone 3, are downregulated by E7.5 or E9.0, respectively [11], [13]. In parallel to H3K9me2 downregulation, H3K27me3, a repressive histone modification providing more plasticity, accumulates in PGCs and finally replaces the H3K9me2 completely at E9.25 [2], [3], [11]. H3K27 trimethylation is catalyzed by Ezh2, a subunit of the polycomb repressive complex 2 (PRC2), and downregulates the expression of typical somatic or differentiation related genes [14], [15]. Ezh2 is subject to phosphorylation at different motifs by the cyclin dependent kinases Cdk1 or Cdk2, which modulate the activity or stability of Ezh2, and thus affect the level of H3K27me3 [16]–[18]. Cdk1/Cyclin B1-mediated phosphorylation of Ezh2 at threonin 487 (pEzh2-T487) disrupts its binding to the other components of PRC2 complex, leading to its inactivation, and therefore to H3K27me3 attenuation [18]. It was previously shown that murine and porcine PGCs, and also PGCs derived in vitro from mouse embryonic stem cells arrest their cell cycle in a G2 phase briefly after their specification [11], [19]–[21]. This phase, which is accompanied by transcriptional silence, may provide time for epigenetic reprogramming. So far, the molecular mechanism coordinating the epigenetic reprogramming and cell cycle prolongation in early PGCs is not clear.

Mad2l2 is a chromatin binding protein involved in both cell cycle control and DNA repair [22]–[24]. Mad2l2 was previously described as an accessory, non-catalytic subunit of the translesion DNA polymerase zeta, and its knockdown led to hypersensitivity towards DNA damage [25], [26]. Mad2l2 appears to function by binding to a diverse spectrum of proteins via its conserved HORMA domain. Several, but not all of these partners bind via the conserved sequence motif PXXXPP [27]. Reported binding partners include Cdh1 and Cdc20, the substrate binding proteins of the APC/C complex, the two translesion polymerases Rev1 and Rev3, the transcription factors Elk-1 and TCF4, the clathrin light chain A, and others [23], [24], [28]–[32]. Accordingly, functions for Mad2l2 were previously claimed in such diverse processes as DNA repair, cell cycle control, and the regulation of gene expression. However, the biological significance of the reported interactions and activities remained unclear due to the lack of appropriate mouse mutants.

In this work we describe a mouse mutant lacking the Mad2l2 gene. Embryos lose PGCs briefly after their specification, and do not proceed in epigenetic reprogramming. We investigated the function of Mad2l2 also by gain- and loss-of-function analysis in fibroblasts, and in biochemical assays. We suggest new functions of Mad2l2 as a regulator of epigenetic reprogramming, which is particularly relevant for primordial germ cells, and therefore required for fertility of males and females.

## Results

### Mad2l2^−/−^ germ cells are lost during early embryogenesis

**Figure 1:**
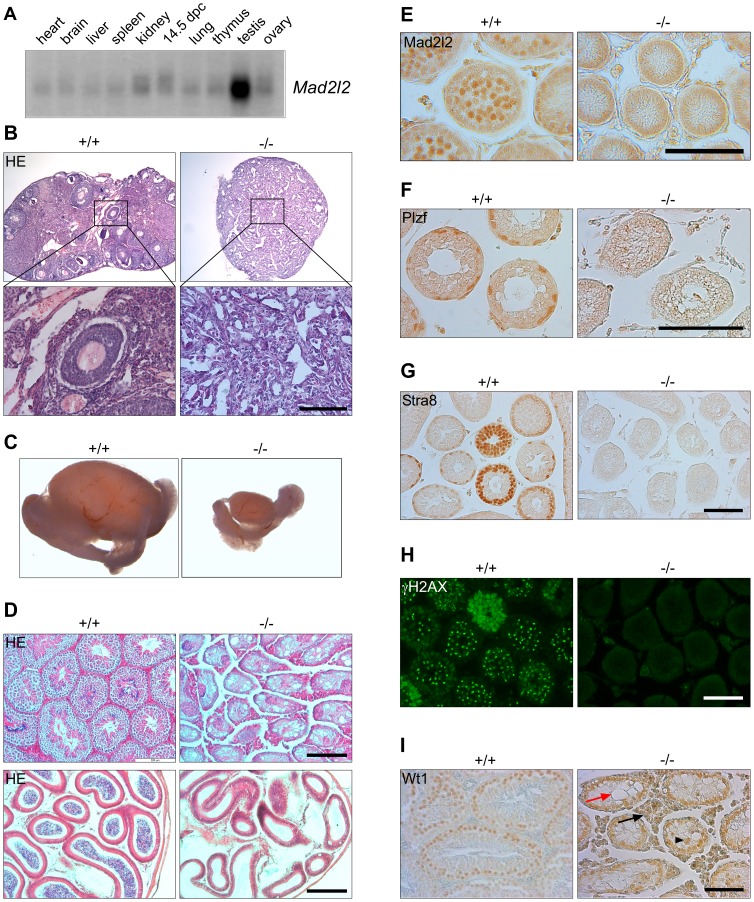
Mad2l2 expression and loss of germ cells from mutant ovaries and testes. (A) Mad2l2 mRNA expression in adult murine organs and E14.5 embryos. For an actin loading control of this northern blot see [74]. (B) Hematoxylin and Eosin (HE) staining of ovaries with low (upper panel) and high (lower panel) magnifications. Mad2l2^−/−^ ovaries (P80) are smaller, and do not contain follicular or germ cells. (C) Testes (P70) are significantly smaller in Mad2l2^−/−^ animals. (D) Morphologic analysis of testes (upper panel) and epididymis (lower panel) by HE staining reveals the absence of germ cells in mutant organs (P70). (E) Mad2l2 protein is expressed in pachytene spermatocytes (P10). (F–H) Mad2l2^−/−^ seminiferous tubules (P14) lack spermatogonial cells as identified by Plzf, pre-meiotic cells as identified by Stra8, and meiotic cells as identified by γH2AX. (I) Mad2l2^−/−^ seminiferous tubules (P70) contain highly vacuolated (red arrow) and miss-localized (arrowhead) Sertoli cells as identified by Wt1. Note hyperplasia of Leydig cells between seminiferous tubules (black arrow). Scale bars in B, E–I, 100 µm, in D, 200 µm.

Low levels of Mad2l2 mRNA are widely expressed in adult and E14.5 embryonic cells, with a particularly high level in testis (Figure 1A). High levels of Mad2l2 protein were detected in pachytene spermatocytes by immunohistochemistry (Figure 1E), while the antibody did not lead to specific signals above background in other tissues, including PGCs. Significant amounts of Mad2l2 RNA were previously detected in E9.5 PGCs by microarray analysis (NCBI database Gene Expression Omnibus GEO; Hayashi et al., 2011).

A conditional knockout of the Mad2l2 gene in embryonic stem cells was generated and ubiquitously active Cre recombinase was introduced through breeding (Figures S1A, B). Heterozygous Mad2l2 mutants were viable, healthy and fertile. Homozygous embryos and postnatal mice were significantly smaller than their littermates, but no morphological abnormalities were observed (Figures S1C–F). Offspring before and after birth appeared in sub-Mendelian ratios, indicating a loss of embryos in midgestation (Table S1). Homozygous males and females were infertile, and gonads were significantly underdeveloped. Ovaries were not formed at all or were small organ rudiments that did not contain ovarian follicles or germ cells (Table S2 and Figure 1B). Such structures may be indicative that some germ cells were present in the gonad during granulosa cell differentiation (Figure 1B). Mutant testes were drastically smaller than control organs of the same age, and seminiferous tubules were devoid of spermatogonial cells (detected by Plzf), pre-meiotic (identified by Stra8) and meiotic cells (detected by γH2AX; Figure 1C,D,F–H) [33]–[36]. Leydig cells appeared hyperplastic, and Sertoli cells, identified by Wt1, were mislocalized and highly vacuolated (Figure 1I) [37], [38]. In summary, finding these deficiencies in both males and females suggested that developmental problems arose earlier during embryogenesis.

**Figure 2:**
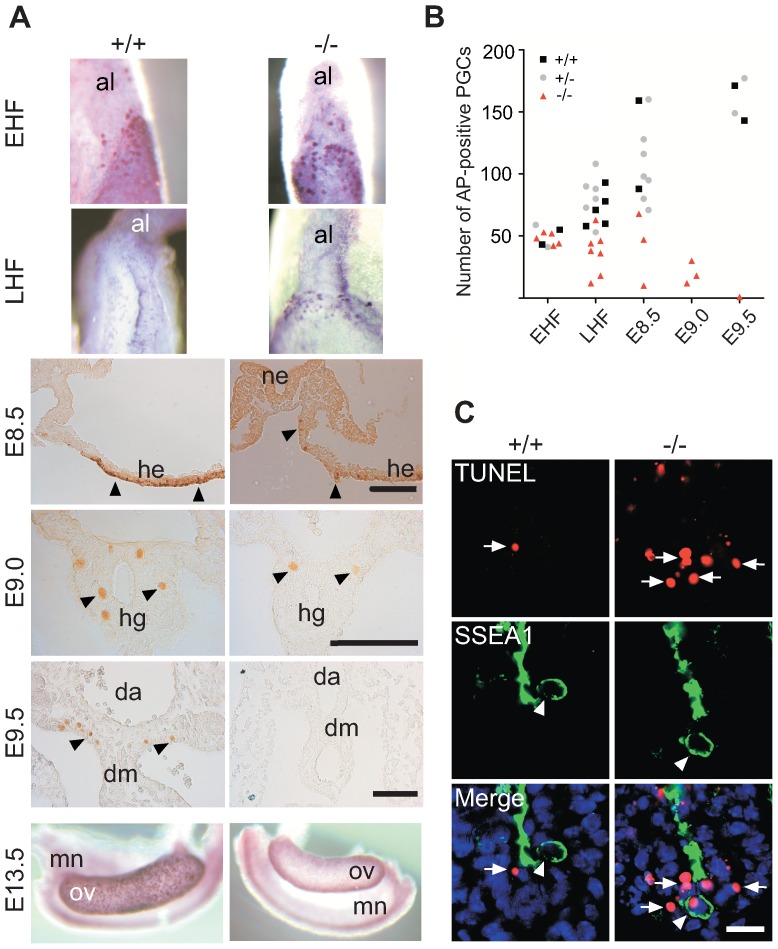
Loss and apoptosis of PGCs early after specification. (A) AP-positive Mad2l2^+/+^ or Mad2l2^−/−^ PGCs were detected in EHF and LHF stages. From E8.5 to E9.5, PGCs were detected by Oct4-immunostaining (arrowheads). At E13.5, Mad2l2^−/−^ ovaries were devoid of germ cells detected by AP staining. Al: allantois; ne: neuroepithelium; he: hindgut epithelium; hg: hindgut; da: dorsal aorta; dm: dorsal mesentery; mn: mesonephros; ov: ovary. Scale bars, 100 µm. (B) Quantification of PGCs detected by AP-staining in different developmental stages. (C) Apoptosis (TUNEL assay) in E9.0 embryo sections of hindgut endoderm. SSEA1-expressing PGCs and apoptotic cells are marked by arrowheads and arrows, respectively. Note the apoptotic PGC in knockout section. Scale bar, 20 µm.

For the determination of PGC numbers, embryos were collected at different time points during their early development, were staged as outlined under experimental procedures, and PGCs were identified by the presence of alkaline phosphatase (AP) or Oct4 (Figure 2A) [39]. At the early head fold (EHF) stage, the numbers of PGCs at the base of the allantois were similar in wild type, heterozygous and homozygous embryos. However, while the number of normal PGCs increased at the late head fold (LHF) stage, the number of Mad2l2^−/−^ PGCs fell behind (Figure 2B). It decreased drastically from E8.5 onward, and at E9.0 only few instead of normally ca. 120 PGCs were found in the hindgut endoderm. At E9.5 and E10.5 Oct4-positive PGCs were no longer detected (Figure 2B). At E8.25, both wild type and remaining mutant PGCs co-expressed Oct4 together with Prdm1, Tcfap2c, and Dppa3, indicating a normal specification of mutant PGCs (Figure S2A,B,D). Oct4 and Sox2 were co-expressed in all wild type PGCs with no exception. In contrast, above 40% of Oct4-positive Mad2l2^−/−^ PGCs did not express Sox2 at E9.0, and thus had either failed to reactivate, or at least to maintain its expression (Figure S2C). Emigration to the dorsal mesentery did not occur, and as a result, gonad primordia at E13.5 were devoid of germ cells (Figure 2A). All E9.0 Mad2l2^−/−^ PGCs had accumulated active, acetylated p53 protein, reflecting an activated stress response and impending apoptosis (Figure S3A) [40]. As judged by the TUNEL assay (See Text S1), some SSEA1-positive PGCs undergoing cell death were detected in E9.0 hindgut endoderm (Figure 2C). In addition, the same territory contained accumulations of SSEA1-negative, apoptotic cells. Based on their size we suspected them to be germ cells having lost already expression of their typical marker, although we could not exclude that they represented mutant somatic cells. In summary, Mad2l2^−/−^ PGCs were specified normally, but their numbers decreased progressively, and no PGCs could be detected in Mad2l2^−/−^ embryos beyond E9.5. This time window correlates with an epigenetic transition of PGCs and cell cycle arrest between E7.5-E9.5 [3], [11].

### Loss of Mad2l2 deficient PGCs is caused by an intrinsic failure

**Figure 3:**
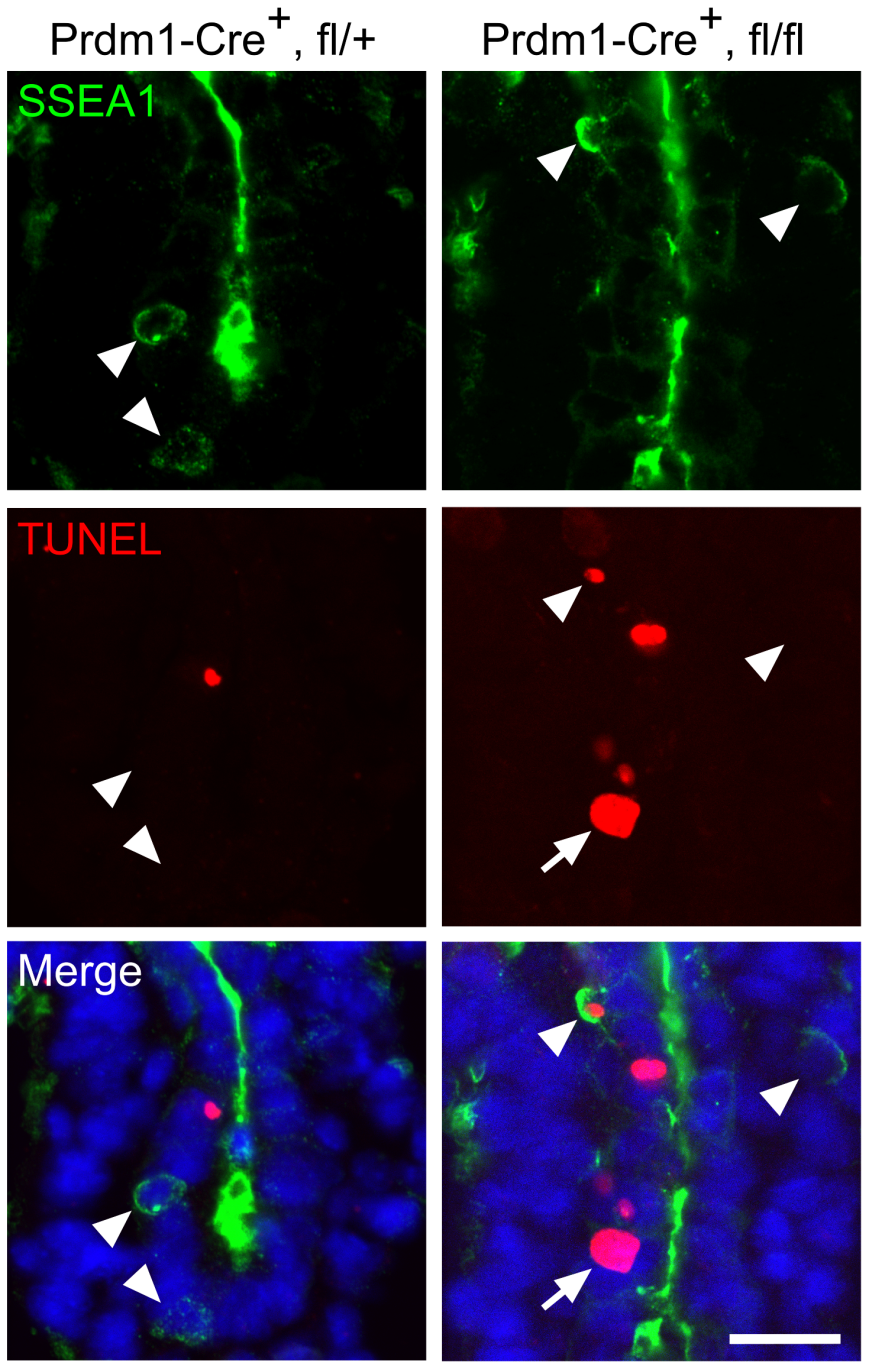
Intrinsic failure of Mad2l2 deficient PGCs. Apoptosis (TUNEL assay) in E8.75 embryo sections of hindgut endoderm after the conditional knockout of Mad2l2 by Prdm1-Cre. SSEA1-expressing PGCs are marked by arrowheads. Note the apoptotic and non-apoptotic PGC in knockout section. Arrow points to an SSEA1-negative apoptotic cell in the conditional knockout section. Scale bar, 20 µm.

Proper development of PGCs relies on their endogenous program as well as on exogenous signals emanating from surrounding somatic cells that support their induction, migration or survival in various organisms [41]–[44]. To address the cause of early PGC loss in Mad2l2 deficient embryos, we employed a Prdm1-Cre mouse line, which would be expected to delete the Mad2l2 gene specifically in nascent PGCs [4]. The TUNEL assay demonstrated apoptosis in SSEA1-positive PGCs of Prdm1-Cre^+^, Mad2l2^fl/fl^ embryos at E8.75 (Figure 3). In addition, TUNEL-positive, SSEA1-negative cells with a high nuclear to cytoplasmic ratio were observed in the hindgut. Also some TUNEL-negative, SSEA1-positive PGCs were found, which is explainable by the incomplete efficiency of Prdm1-Cre mediated deletion, although the actual recombination could not be confirmed here for the few available cells [4]. In contrast, no apoptosis was observed in Prdm1-Cre^+^, Mad2l2^fl/+^ PGCs of the same age, excluding toxic effect of Cre recombinase on PGCs [45]. Together, these findings demonstrate that Mad2l2 deficient PGCs did not survive even in a wild type somatic environment. Since Mad2l2 is the subunit of a repair DNA polymerase, we asked if Mad2l2 deficient PGCs are affected by DNA damage. We applied an antibody detecting phosphorylated ATM/ATR substrates (pATM/ATR-S) including Chk1, Chk2, and MDM2, as well as specific antibodies against pChk1 and pChk2, respectively. No double-positive PGCs were detected in either wild type or knockout embryos in such staining (Figure S3B–D). Together, these observations indicate that Mad2l2 deficient PGCs are not lost due to DNA damage.

### Mad2l2 deficiency affects epigenetic reprogramming of histones and cell cycle arrest in PGCs

**Figure 4:**
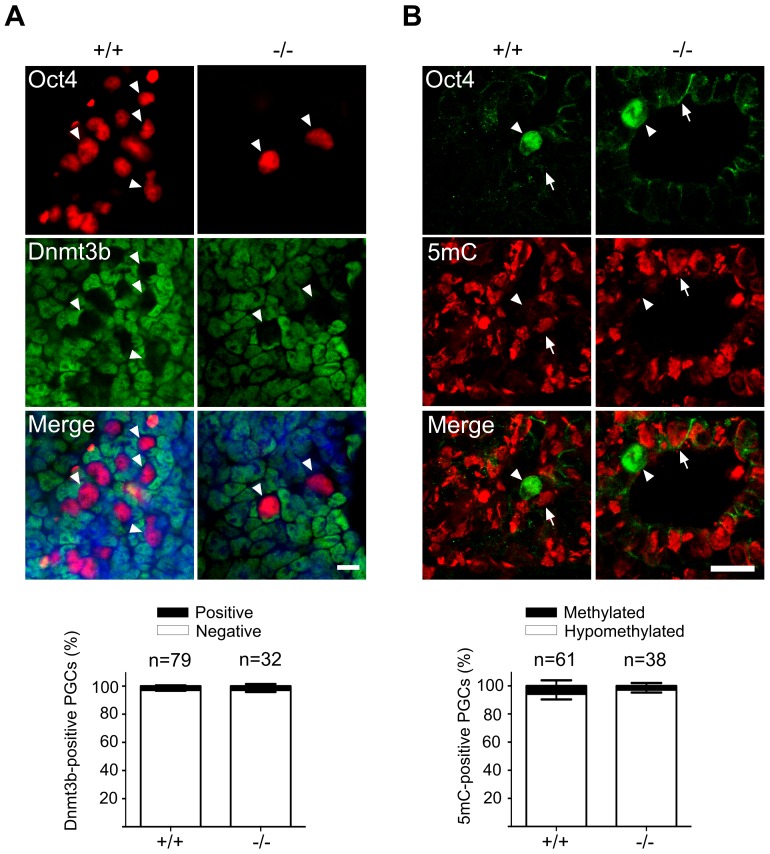
Normal DNA demethylation in Mad2l2 deficient PGCs. (A) Whole mount staining of E9.0 embryos (upper panel) and related quantification (lower panel) shows a normal down regulation of Dnmt3b DNA methyltransferase. (B) Immunohistochemistry analysis of embryo sections at E9.0 represents a normal DNA demethylation of both wild type and knockout PGCs (arrowheads). The arrow points to a somatic cell with a high DNA methylation level. “n” represents the total number of PGCs counted in three different embryos per genotype. The data are means ± SD.

Immediately after their induction in the epiblast, PGCs begin to undergo massive epigenetic reprogramming with regard to both DNA and histone modifications. The genome-wide demethylation of the DNA in PGCs is partially due to a downregulation DNA methyltransferases, which is accompanied by loss of cytidine methylation. To address the epigenetic reprogramming in Mad2l2^−/−^ PGCs, first we performed whole mount staining (See Text S1) against Dnmt3b DNA methyltransferase. Both wild type and Mad2l2 deficient PGCs suppressed Dnmt3b expression (Figure 4A). Immunohistochemistry analysis of DNA methylation showed loss of the 5-methylcytosine (5 mC) at E9.0 in both wild type and knockout sections (Figure 4B). These observations seem to indicate that DNA hypomethylation had been properly initiated and progressed in the absence of Mad2l2.

**Figure 5:**
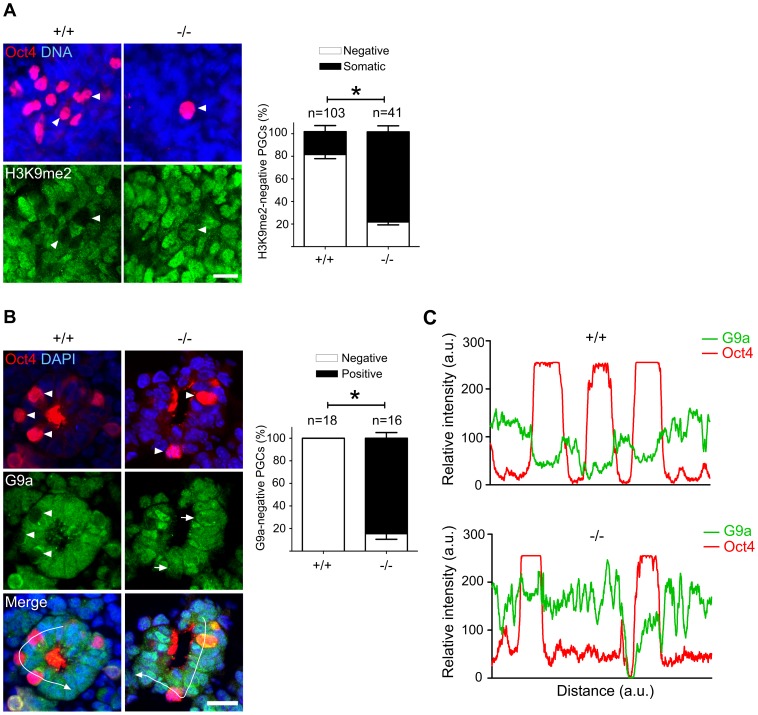
Majority of Mad2l2 deficient PGCs fail to downregulate H3K9me2. (A) At E9.0, the majority of Mad2l2^+/+^ PGCs had suppressed successfully H3K9me2 (arrowheads), while many Mad2l2^−/−^ PGCs (arrowhead) maintained this epigenetic mark at levels similar to neighboring somatic cells. Right panel: quantification of H3K9me2-negative PGCs (white bars), and of PGCs expressing H3K9me2 at a similar level to their neighboring somatic cells (black bars). “n” represents total number of PGCs counted at least in three embryos per genotype. Data are means ± SD. Asterisk represents *P*≤0.05. Scale bar, 20 µm. (B) G9a expression was absent from all Mad2l2^+/+^ PGCs at E9.0 (arrowheads, 0%, 0/18). Most Mad2l2^−/−^ PGCs were positive for G9a (arrowheads, 87%, 14/16). Right panel: quantification of G9a-negative (white bars) and G9a-positive (black bar) PGCs. Data are means ± SD. Asterisk represents *P*≤0.01. Scale bar, 20 µm. (C) Line-scan profile of relative intensity of G9a and Oct4 fluorescent signals in (B).

In PGCs, the repressive histone H3K9me2 should become downregulated during the cell cycle arrest between E7.5 and E9.5. A comparison of stage-matched E9.0 embryos revealed that the majority of mutant, Oct4-positive PGCs had not downregulated H3K9me2, while wild type PGCs mostly had lost this histone modification (Figure 5A). Correspondingly, also G9a and GLP, two H3K9 methyltransferases, were still found in mutant, but not in wild type PGCs (Figure 5B,C; S4A,B).

**Figure 6:**
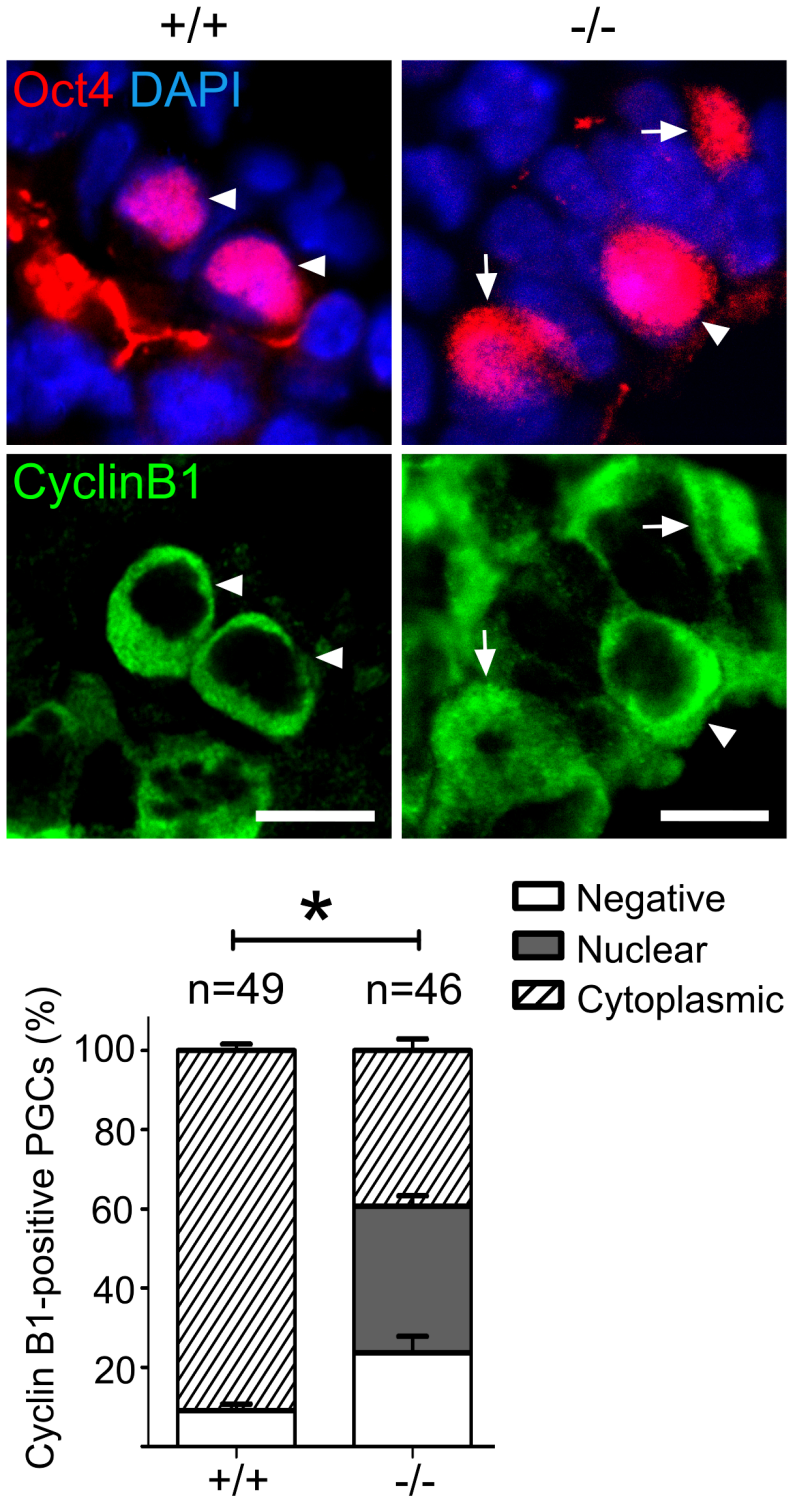
Mad2l2 deficiency affects the cell cycle in PGCs. Immunohistochemistry on transverse sections of E9.0 embryos. PGCs were identified by Oct4 (upper panel). Cytoplasmic staining of Cyclin B1 in Mad2l2^+/+^ PGCs (arrowheads, 90.9%) indicated that the majority had arrested in the G2 phase of their cycle (lower panel). Mad2l2^−/−^ PGCs expressed Cyclin B1 in the nucleus (37%, arrows), in the cytoplasm (39.3%, arrowhead), or were negative (23.66%), suggesting active cycling. “n” represents total number of PGCs counted in three embryos of each genotype. Data are means ± SD. Asterisk indicates *P*≤0.01. Scale bars, 10 µm.

Addressing the cell cycle profile of PGCs, we confirmed a cytoplasmic localization of Cyclin B1 in the majority of wild type PGCs on E9.0, indicating that they were in the G2 phase of the cell cycle (Figure 6) [11]. In Oct4-positive Mad2l2^−/−^ PGCs, on the other hand, the Cyclin B1 protein was either localized in the nucleus, in the cytoplasm or not present at all (Figure 6). Thus, it appeared that mutant PGCs did not arrest in G2 phase of their cell cycle.

**Figure 7:**
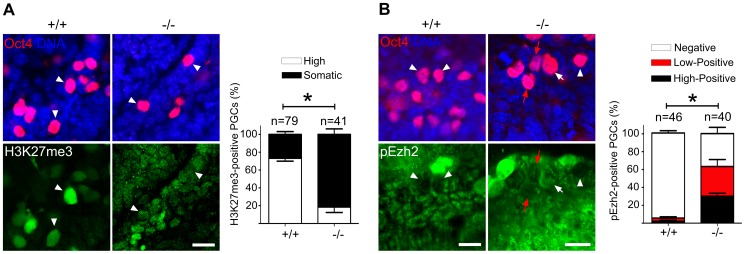
Majority of Mad2l2 deficient PGCs fail to upregulate H3K27me3. (A) The majority of Mad2l2^+/+^ PGCs had upregulated H3K27me3 by E9.0 (arrowheads), whereas many Mad2l2^−/−^ PGCs (arrowheads) failed to upregulate above the basal level in somatic cells. Data were obtained by whole mount staining for Oct4 and H3K27me3. Right panel: Quantification of PGCs strongly positive for H3K27me3 (white bars). Black bars show the percentage of PGCs that express H3K27me3 at a level similar to their neighboring somatic cells. (B) The majority of Mad2l2^+/+^ PGCs suppress the phosphorylation of Ezh2 (pEzh2; arrowheads), whereas above 60% of Mad2l2^−/−^ PGCs failed to downregulate pEzh2 (white arrow indicates highly positive, and red arrows point to low-positive PGCs). Data were obtained by whole mount staining for Oct4 and pEzh2 at E8.5. Right panel: quantification of pEzh2-negative PGCs (white bars). Black and red bars show the percentage of PGCs with high or low levels of pEzh2, respectively. In (A) and (B), “n” represents total number of PGCs counted at least in three embryos per genotype. Data are means ± SD. Asterisk represents *P*≤0.05 in both (A) and (B). Scale bar, 20 µm.

A highly elevated, global H3K27me3 modification could be confirmed for the majority of wild type PGCs, while levels in Mad2l2^−/−^ PGCs were mostly indistinguishable from surrounding somatic cells (Figure 7A). Ezh2, the relevant methyltransferase for residue K27 of histone 3, is expressed in PGCs at a similar level to that of neighboring somatic cells, at least during their specification period [46]. However, we observed that the inactivation of Ezh2 was completely suppressed in the majority of wild type PGCs at E8.5, while above 60% of knockout PGCs contained high or low levels of such inactive Ezh2 protein (Figure 7B). Thus, a significant portion of the Mad2l2^−/−^ PGCs failed to acquire an epigenetic status dominated by H3K27me3, probably due to presence of inactive phosphorylated Ezh2.

### Mad2l2 affects the status of histone modifications and cell cycle in fibroblasts

**Figure 8:**
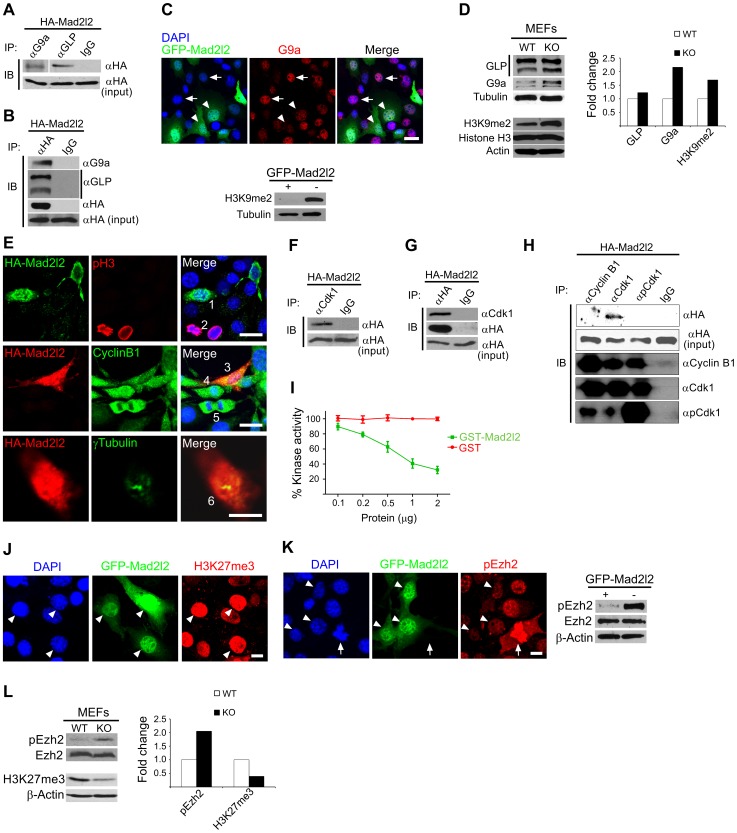
Analysis of Mad2l2 function in fibroblasts. (A) Protein extracts from HA-Mad2l2 transfected NIH3T3 cells were co-immunoprecipitated (IP) by antibodies against G9a, GLP, or IgG (as negative control). Immunoblotting (IB) was performed on 20% of gel-separated immunoprecipitates (upper blot), or 1% input (lower blot) by using anti-HA antibody. (B) Reciprocally, the same protein extract was co-immunoprecipitated (IP) by antibodies against the HA-tag, or IgG (as negative control). Immunoblotting (IB) was performed on 20% of the immunoprecipitates (upper blots), or 1% input (lower blot) by using anti-G9a, anti-GLP, or anti-HA antibodies. (C) Immunocytochemistry detects a downregulation of G9a in GFP-Mad2l2 over-expressing NIH3T3 cells (arrowheads) in comparison to untransfected cells (arrows). The lower panel shows a western blot analysis of H3K9me2 levels in GFP-Mad2l2 over-expressing, FACS-sorted NIH3T3 cells versus non-transfected NIH3T3 cells. Note the efficient downregulation of H3K9me2 by Mad2l2 overexpression. (D) A representative western blot analysis of GLP, G9a, H3K9me2 and Histone H3 levels in wild type versus knockout MEFs (left panel) and quantification of the western blot bands normalized to tubulin or actin signals (right panel). (E) The effect of Mad2l2 on cell cycle parameters. HA-Mad2l2 transfected NIH3T3 fibroblasts never expressed pH 3 (0%, 0/70; e.g. cell number #1, upper panel), and always displayed Cyclin B1 in the cytoplasm (100%, 40/40; #3, middle panel). Some of the non-transfected cells entered the mitotic prophase (#2, #4) or anaphase (#5), and displayed nuclear pH 3 (#2) or nuclear Cyclin B1 (#4, #5). HA-Mad2l2 expressing cells displayed two unseparated centrosomes detectable by γTubulin (100%, 7/7; #6, lower panel). Scale bars, 20 µm (upper and middle panels), 10 µm (lower panel). (F–G) Reciprocal co-immunoprecipitation of HA-Mad2l2 and Cdk1 from HA-Mad2l2 over-expressing protein extract, using either anti-HA or anti-Cdk1 antibodies. 50% of the immunoprecipitates, or 1.5% of total cell lysate (input) were loaded. (H) Cdk1 antibody co-immunoprecipitated HA-Mad2l2 from transfected NIH3T3 cells, but not antibodies against Cyclin B1, pCdk1, and rabbit IgG. 50% of the immunoprecipitates, or 1.5% of total cell lysate (input) were loaded. (I) Recombinant GST-Mad2l2 attenuates the kinase activity of Cdk1-Cyclin B1 (2.5 mUnits) in vitro, while GST alone is not effective. Mean values of three independent experiments with duplicate measurements, and standard deviations are indicated. (J) Immunocytochemistry demonstrates the upregulation of H3K27me3 in GFP-Mad2l2 over-expressing NIH3T3 cells (arrowheads). (K) Immunocytochemistry analysis shows suppression of phosphorylation on Ezh2 at T487 (white arrowhead) in comparison to surrounding, untransfected interphase cells. The highest level of pEzh2 was detected in mitotic cell with high level of Cdk1 activity (arrow). The right panel shows a western blot analysis of pEzh2 and Ezh2 levels in FACS-sorted, GFP-Mad2l2 over-expressing NIH3T3 cells and untransfected controls. (L) A representative western blot analysis of pEzh2, Ezh2, H3K27me3, and actin levels in wild type versus knockout MEFs (left panel) and quantification of the western blot bands normalized to actin signal (right panel). Note the inhibition of Ezh2 by phosphorylation, and the concomitant decrease of H3K27me3 in the absence of Mad2l2.

The number of early PGCs is too small for biochemical and transfection approaches. Therefore, we performed a set of experiments in fibroblasts with the intention to provide evidence for a function of Mad2l2 in epigenetic and cell cycle regulation. Since the Mad2l2 protein contains a protein-binding HORMA domain Co-immunoprecipitation was applied to identify Mad2l2 interacting partners related to histone modifications (See Text S1). First, to explore a physical interaction between Mad2l2 and G9a or GLP, NIH3T3 fibroblasts were transfected with a plasmid encoding HA-Mad2l2 (See Text S1). Co-immunoprecipitation of NIH3T3 protein extract with anti-G9a, anti-GLP or anti-HA antibodies demonstrated that Mad2l2 interacts with both methyltransferases (Figure 8A, B). Transfection of NIH3T3 cells with a vector encoding a GFP-fused Mad2l2 protein showed that G9a mRNA levels were specifically downregulated in the presence of GFP-Mad2l2 (Figures S5A). G9a protein levels were always low in Mad2l2-GFP transfected cells, while untransfected cells had either high or low levels (Figures 8C). Correspondingly, the level of H3K9me2 became completely suppressed in transfected cells (Figure 8C), while levels of H3K4me2, an unrelated histone modification, remained unaffected (Figure S5B). For the analysis of loss-of-function conditions Mad2l2 deficient MEFs were prepared, and elevated levels of G9a and H3K9me2 were observed (Figure 8D). Together, these findings indicate a negative correlation between the presence of Mad2l2 and the expression and activity of the methyltransferase G9a.

To test whether ectopic expression of Mad2l2 can arrest the cell cycle, NIH3T3 cells were transfected with a HA-Mad2l2 encoding vector. Expressing cells did not enter mitosis, as evident by the complete absence of pH 3 or Cyclin B1 from nuclei, as well as the presence of unseparated centrosomes (Figure 8E) [47], [48]. Several pathways regulating the entry into mitosis converge at the cyclin dependent kinase 1 (Cdk1), which needs to be dephosphorylated and associated with phosporylated Cyclin B1 to be active [49], [50]. We hypothesized that Mad2l2 might interact physically with Cdk1 or Cyclin B1 to regulate the G2/M transition. Protein lysate from HA-Mad2l2 transfected NIH3T3 cells was precipitated with antibodies against Cdk1, pCdk1 (phosphorylated Cdk1), Cyclin B1, and the HA-tag. Co-precipitate analysis revealed a physical interaction of Mad2l2 with Cdk1, but not pCdk1 or Cyclin B1 (Figure 8F–H). We then looked for a regulatory effect of Mad2l2 on the kinase activity of Cdk1/Cyclin B1 in an in vitro assay (See Text S1), containing recombinant GST-Mad2l2, Cyclin B1 and Cdk1, as well as the specific substrate Cdc7 [51]. GST-Mad2l2, but not GST alone could specifically attenuate the kinase activity of Cdk1-Cyclin B1 in a concentration-dependent manner (Figure 8I). Together, our experiments suggest that the ectopic presence of Mad2l2 prolongs the cell cycle.

To address whether Mad2l2 can principally be involved in H3K27me3 upregulation, gain-of-function experiments with a GFP-Mad2l2 fusion protein were performed in NIH3T3 cells. Immunocytochemistry showed a very high level of H3K27me3 in all GFP-positive cells, while surrounding untransfected cells had mostly low levels, with some exceptions possibly dependent on the state of their cell cycle (Figure 8J). Given the inhibitory function of Mad2l2 on the kinase activity of Cdk1, we asked if it might attenuate the inhibitory phosphorylation of Ezh2 (Figure 8K, L). The highest level of pEzh2 was observed in mitotic cells correlating with the highest activity of Cdk1/Cyclin B1 (Figure 8K) [18]. In contrast, Mad2l2 over-expressing cells showed the lowest level of pEzh2, even less than that in untransfected interphase cells (Figure 8K). Consistently, western blot analysis confirmed the drastic suppression of pEzh2 in Mad2l2 over-expressing FACS-sorted fibroblasts, while the overall level of Ezh2 itself remained unchanged (Figure 8K). The loss-of-function situation was analyzed in Mad2l2 deficient MEFs, which showed an increased level of pEzh2, while the amount of H3K27me3 was decreased (Figure 8L). Apparently, here the Cdk1/Cyclin B1 was active, and could phosphorylate and thereby inactivate Ezh2. Our analysis of fibroblasts and of a cell free system demonstrate the capacity of Mad2l2 to suppress the kinase activity of Cdk1/Cyclin B1, and thus to support the activity of Ezh2 and by that promote the tri-methylation of histone 3 on K27.

## Discussion

Several mutations are known to affect or terminate the development of PGCs (for review see [44]). In principal, every step proved to be sensible, particularly the primary induction by BMP signaling, the early specification, the migration to the developing gonad, and the pre- or postnatal oogenesis or spermatogenesis. The early BMP response genes, Prdm1 and Prdm14, are crucial for PGC specification directly after induction, where numbers of mutant PGCs are drastically reduced already on E8.0, and only few mutant PGCs survive to E9.5 [4], [5]. Similar kinetics for PGC loss were observed in mice lacking the transcription factor Tcfap2c, which mostly phenocopy the Prdm1^−/−^ mice [52]. A slightly later timing, shifted by about one day, was found for the Mad2l2 mutants in our study. Although embryos at EHF stage were relatively small, they harbored stage-adequate numbers of PGCs expressing Prdm1 and the commitment markers Dppa3 and Tcfap2c arguing for a normal specification in the epiblast. A reduction of PGC numbers was observed in the LHF stage, and there was no survival beyond E9.5. At this point of development, PGCs would normally have undergone a major epigenetic reprogramming, would recover from their cell cycle arrest, and resume transcription. This timing suggests a failure of epigenetic reprogramming and cell cycle arrest in Mad2l2^−/−^ PGCs. In principle, it is conceivable that wrongly developed PGCs might either revert to a somatic fate, or undergo apoptosis. PGCs are lost without evidence for apoptosis in mutants of the Prdm1, the Prdm14, and the Tcfap2c gene, whereas mutations in the Oct4, the Kit and the Mad2l2 genes remove wrongly programmed PGCs by apoptosis [4], [5], [52]–[54]. Somatic Mad2l2^−/−^ cells apparently do not rely on a specific epigenetic reprogramming and cell cycle arrest, and at least some Mad2l2-deficient mice develop normally and live until adulthood. Still, mutants are born in sub-Mendelian ratio and adults are usually smaller, as is the case in many mutant mice. Together, this points to a highly specialized function of Mad2l2 in the unique development of germ cells, but does not exclude lower penetrance effects in somatic cells.

H3K9 methylation is critical for formation of heterochromatin and transcriptional silencing. At the onset of PGC development, H3K9me2 is the dominant epigenetic mark in the genome of embryonic cells [3], [11]. This modification requires the activity of the two methyltransferases G9a and GLP [55]. G9a, the major mammalian H3K9 methyltransferase, plays a critical role in germ cell development, particularly in gametogenesis. The specific deletion of G9a in PGCs after E9.5 leads to germ cell loss during the meiotic prophase, and thus to sterility of both males and females [56]. During the S phase of the cell cycle, G9a binds to DNA methyltransferase DNMT1 and loads on to the DNA at replication foci, ensuring a coordination of DNA methylation and H3K9 methylation in heterochromatin regions [57]. Nascent PGCs leave asynchronously the S phase of their cycle and enter G2 at around E8.0. At this time, the de novo methylation of the daughter chromatin is suppressed, and both Prdm1 and Prdm14 were suggested to be involved [58], [59]. In parallel, the maintained activity of histone demethylases like Jmjd1a erases further the remaining H3K9me2 [60]. Our results indicate that similar to Prdm14 deficient PGCs, the majority of Mad2l2^−/−^ PGCs fail to suppress H3K9me2. The maintenance of a high H3K9me2 level in Prdm14 mutant PGCs was attributed to a failure in downregulation of GLP. Released from repression by genome-wide H3K9me2, PGCs repress RNA Pol-II dependent de novo transcription until they acquire the alternative repressive histone mark, H3K27me3. This probably ensures the maintenance of separate PGC and somatic programs, established previously via combinational functions of Prdm1, Prdm14, and Tcfap2c [61]. A significant portion, but not all, of the Mad2l2^−/−^ PGCs failed to proceed with their epigenetic reprogramming, as it is the case in Prdm14 mutant PGCs. Obviously, shortly before their elimination around E9.0, the Mad2l2^−/−^ PGCs represent a heterogeneous population with respect to their transcriptional and epigenetic status. Thus, Mad2l2 is absolutely essential for the development of PGCs.

We observed that Mad2l2 suppresses G9a on the level of gene expression, which could be related to its ability to interact with transcription factors [29], [32]. The binding of Mad2l2 to the two histone methyltransferases G9a and GLP was previously identified in a systematic analysis of human protein complexes, and represented a first hint for an involvement of Mad2l2 in the generation of epigenetic modifications [62]. We confirmed this evidence by co-immunoprecipitation of both G9a and GLP with HA-Mad2l2 from transfected fibroblasts, where the level of H3K9me2 was significantly downregulated. Noteworthy, both G9a (PXXXPP) and GLP (PXXXyP) have the sequence motif suggested to be responsible for Mad2l2 binding [27]. G9a and GLP form homo- and heteromeric complexes in vitro, which are necessary for histone methyltransferase activity [13], [55]. Indeed, several proteins, bind to G9a or GLP, and alter their activities [63], [64]. Among those is Prdm1, which binds to G9a and recruits it to assemble silent chromatin [65]. Similarly, the direct interaction between Mad2l2 and G9a or GLP may disrupt formation of the G9a-GLP active heterodimer complex, and thus suppress the methylation of histone 3. Supportive evidence for such an inhibitory binding comes from the negative correlation between Mad2l2 and H3K9me2 levels in PGCs (Fig. 5A) and fibroblasts (Fig. 8D). However, the actual significance of the observed protein-protein interactions needs further investigation.

**Figure 9:**
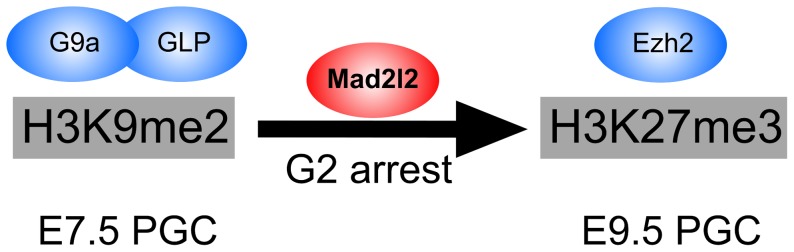
The role of Mad2l2 in epigenetic reprogramming and G2 arrest in PGCs. The model describes the function of Mad2l2 in the coordination of cell cycle arrest and the epigenetic transition of PGCs from H3K9me2 state at E7.5 to H3K27me3 state at E9.5. In the absence of Mad2l2, a majority of PGCs fail to either downregulate H3K9me2, or upregulate H3K27me3, or arrest in G2 phase of their cell cycle.

Cdk1 is a regulatory kinase of central importance for several processes, in particular also in cell cycle control and in epigenetic reprogramming [66], [67]. Our study in transfected fibroblasts and in a cell-free system suggests that Mad2l2 can bind directly to dephosporylated Cdk1, and thus inhibit its kinase activity. Possibly this interaction involves the Cdk1 sequence PXXXPy, which is related to the previously identified Mad2l2 binding motif PXXXPP [27]. The entry into mitosis is mediated by a complex network of proteins that finally activate the Cdk1-Cyclin B1 complex [50]. One of the first functions of Cdk1-Cyclin B1 is the phosphorylation and therefore disruption of Eg5, a protein involved in centrosome adhesion [68]. Overexpression of Mad2l2 abrogated centrosome separation, and caused a cell cycle arrest at the G2 phase. Dephosphorylated Cdk1 in association with phosphorylated Cyclin B1 translocate to the nucleus and initiates prophase by the phosphorylation of a variety of substrates [50]. Thus, via direct binding to Cdk1, Mad2l2 would have the capacity to inhibit Cdk1-Cyclin B1 complex formation, and thus to block the entry into mitosis. Inhibition and/or disruption of the Cdk1-Cyclin B1 complex through direct interaction were previously also observed for Gadd45 proteins, stress factors implicated in the activation of the G2/M DNA damage checkpoint [51], [69], [70]. Previous analyses of Mad2l2 had indicated inhibitory interactions with Cdh1, and possibly also with Cdc20 [23], [24]. These proteins would normally exert their function only after the onset of mitosis, either as part of the spindle assembly checkpoint, or as the substrate recognizing protein of the APC/C protein ubiquitination complex, respectively. However, early knockout PGCs divide relatively normal and only fail to arrest in the G2 phase. Therefore, it is less likely that Mad2l2 functions in mitosis of PGCs via binding to Cdh1, or Cdc20. Overexpression in fibroblasts indicated the possibility that Mad2l2 can be involved in a G2 arrest. This might correlate with the G2 arrest, which coincides with the epigenetic transition of PGCs from a H3K9me2 to a H3K27me3 configuration, and with the timing of PGC loss in Mad2l2 mutants. Among the many functions of the widely distributed kinase Cdk1 is the inhibition of the histone 3 methyltransferase Ezh2 by phosphorylation [66], [67]. Our analysis in fibroblasts indicates that Mad2l2 can interfere with this inactivation, and thus in effect, promote the activation of Ezh2. Consequently, we observed an increase of H3K27me3 levels upon overexpression of Mad2l2. Our data do not allow at present to decide if the primary defect in knockout PGCs lies in the regulation of the cell cycle, if the epigenetic failure precedes misregulation of the cycle, or if the two tightly coupled processes are not separable. Nevertheless, the outcome is that Mad2l2 mutated PGCs are not able to make the developmental transition from E7.5 to E9.5, and are quickly eliminated from the embryo (Figure 9). Thus, Mad2l2 is absolutely required for the development of PGCs, and thus for fertility.

While this manuscript was under revision, a related set of data was published demonstrating the necessity of Mad2l2 for PGC maintenance [71]. However, detailed characterization of knockout PGCs and the mechanism by which Mad2l2 may function were not studied.

## Materials and Methods

### Ethics statement

All animal works have been conducted according to relevant national and international guidelines.

### Gene targeting strategy

Genomic sequences were amplified from a 129 strain mouse PAC clone. The vector was assembled using the recombineering protocol and materials as described (see Figure S1; [72]. The loxP sites were introduced 113 bp upstream of the first coding exon, and 20 bp dowstream of the last exon, deleting finally a region of 5330 bp. The vector was introduced into MPI-II ES cells, which were selected with G418 and Ganciclovir. Cells with homologous recombination were aggregated with morula-stage embryos. The Mad2l2 gene was inactivated by crossing of heterozygote mice with CMV-Cre mice [73], and then breeding to homozygocity. Genotyping was performed using the primers

#1 (GCTCTTATTGCCTTGACATGTGGCTGC),

#2 (GGACACTCAGTTCTGGAAAGGCTGG), and

#3 (CTGCAGCCCAATTCCGATCATATTCAATAAC).

### Embryos

The day of the vaginal plug was taken as E0.5, and embryos were dissected accordingly. Embryos were staged [11] by corresponding time and morphology as follows: before E8.0 (EHF), E8.0 (LHF), E8.25 (less than 5 somites), E8.5 (before turning, 6 to 8 somites), E8.75 (turning embryos, 10 to 12 somites), E9.0, (after turning, 14 to 18 somites, with only the first branchial arch obvious, and with open otic vesicles, E9.5 (two branchial arches, closed otic vesicles, 20–24 somites).

### Antibodies

The following antibodies were used. Rabbit anti-Cyclin B1 (Sigma-Aldrich), 1∶100; mouse anti-phospho-Histone H3 (ser10; Cell Signaling), 1∶200; rat anti-HA (Roche), 1∶100; mouse anti-γTubulin (Abcam), 1∶200; mouse anti-Cdk1 (Santa Cruz), 1∶50; rabbit anti-pCdk1 (Cell Signaling), 1∶50; mouse anti-Oct4 (BD), 1∶100; rabbit anti-Oct4 (Abcam), 1∶100; mouse anti-SSEA1 (Santa Cruz), 1∶100; rabbit anti-Nanog (abcam), 1∶100; rabbit anti-Sox2 (Millipore), 1∶200; rabbit anti-H3K9me2 (Upstate) 1∶100; and (Millipore), 1∶100; rabbit anti-G9a (Cell Signaling), 1∶25; mouse anti-GLP (Abcam), 1∶50; rabbit anti-Mad2l2 (Abcam), 1∶100; mouse anti-γH2AX (Millipore), 1∶200; rabbit anti-pChk2 (Cell Signaling), 1∶200; mouse anti-Vimentin (gift of M. Osborn), 1∶100; rabbit anti-WT1 (Abcam), 1∶1000; rabbit anti-Ezh2 (Cell Signaling), 1∶2000; rabbit anti-pEzh2 T487 (Epitomics), 1∶1000; rabbit anti-H3K4me2 (Active Motif), 1∶100; rabbit anti-H3K27me3 (Active Motif), 1∶100; rabbit anti-Dppa3 (abcam), 1∶500; rabbit anti-Stra8 (abcam), 1∶2000; rabbit anti-Plzf (abcam), 1∶100; rabbit anti-Dnmt3b (abcam), 1∶100; rabbit anti-Tcfap2c (Santa Cruz), 1∶100; mouse anti-5mC (abcam), 1∶200.

### GST-Mad2l2 preparation

GST-fused Mad2l2 protein was expressed in and purified from *E. coli*. Full length Mad2l2 cDNA was cloned in frame with the N-terminal GST-tag into the pGEX-KT vector. Expression was induced by the addition of 1 mM IPTG (isopropyl-β-D-thiogalactopyranoside, Sigma). Bacterial cells were harvested; proteins were lysed on ice in 50 mM Tris, pH 7.5, 500 mM NaCl, 2 mM EDTA, 5 mM DTT, 10% glycerol, freshly added 1 mM PMSF and Complete EDTA-free protease inhibitor cocktail tablet (Roche). Glutathione Sepharose 4B (Amersham Biosciences) was used to purify the GST-fused protein. The elution was done twice, each time with 2 ml elution buffer (500 mM Tris, pH 8.0, 100 mM Glutathione supplemented with protease inhibitor). The protein was dialyzed in dialysis buffer (20 mM Tris-Cl pH 7.5) using a dialysis cassettes (Pierce) at 4°C overnight. The protein concentrations were measured and determined according to the standard curve.

### Kinase assay

Kinase activity of Cdk1-cyclin B1 was analyzed using purified, recombinant proteins (CycLex), and a human Cdc7 peptide as substrate, applying an assay system from CycLex [51]. To test effect of Mad2l2 on kinase activity of Cdk1-Cyclin B1, dilutions of GST-Mad2l2 or GST alone protein were incubated for 15 min at 37°C with 12.5 mUnits of recombinant kinase. These protein mixes were individually given into substrate-coated wells, and incubated for 45 min at 37°C. For detection of phospho-Cdc7 a specific monoclonal antibody (TK-3H7) and HRP-conjugated anti-mouse IgG was applied, and the absorbance at 450 nm was measured.

## Supporting Information

Figure S1 Generation and general characterization of Mad2l2 knockout mouse line. (A) Gene targeting strategy. B = Bgl1, D = Dral recognition sites. Arrows #1, 2, 3 indicate genotyping primers. (B) Confirmation of homologous recombination in Mad2l2 locus by Southern blotting of ES cells DNA. (C) Size reduction of Mad2l2 mutants. E12.5, E17.5 embryos and newborn mice on postnatal day 7 (P7) are shown. (D) Postnatal development of Mad2l2^−/−^ mutants remains retarded. (E) Comparison of adult animals' weight shows a significant reduction in knockouts. Right graph: the average weight represented as mean ± SD of at lease three animals per each genotype. Asterisk indicates *P*≤0.01.
(TIFF)

Figure S2 Expression of PGC-specific markers. (A,B,D) Both wild type and knockout PGCs express Prdm1, Dppa3, and Tcfap2c at E8.5. At least 50 PGCs per each genotype were analyzed. Scale bars: 20 µm. (C) Sox2 expression characterizes all Mad2l2^+/+^ PGCs at E9.0 (100%, 17/17). Many Mad2l2^−/−^ PGCs of the same stage were negative for Sox2 (44%, 8/18; arrows; *P*≤0.05), or were only weakly positive (arrowheads).
(TIFF)

Figure S3 No activation of DNA damage response was observed in apoptotic Mad2l2^−/−^ PGCs. (A) Mad2l2^−/−^ PGCs expressed active, acetylated p53 (arrowheads, 100%, 6/6). PGCs were identified by Oct4 immunohistochemistry on transverse sections of E9.0 embryos (arrowheads). (B) No Oct4- and phospho ATM/ATR substrate-double positive PGCs were detected in Mad2l2^−/−^ embryo section at E9.0 (arrowheads). Arrow indicates a positive somatic cell implying the proper staining. (C, D) No Oct4- and phospho-Chk1 (C) or phospho-Chk2 (D) double positive Mad2l2^−/−^ PGCs were detected at E9.0 (arrowheads). In contrast, occasionally, some somatic cells showed expression of these active DNA damage response markers (arrows). Scale bars: A and C, 20 µm, B and D, 10 µm.
(TIFF)

Figure S4 Mad2l2 deficient PGCs fail to downregulate GLP. (A) GLP expression was absent from all Mad2l2^+/+^ PGCs at E9.0 (arrowheads, 0%, 0/18). Most Mad2l2^−/−^ PGCs were positive for GLP (arrowheads, 87.5%, 14/16; *P*≤0.05). (B) Line-scan profile of relative intensity of GLP and Oct4 fluorescent signals in (A).
(TIF)

Figure S5 Analysis of Mad2l2 function in fibroblasts. (A) qRT-PCR analysis of G9a expression in FACS sorted NIH3T3 cells. GFP-Mad2l2 overexpression downregulates the G9a level to around half the value in non-transfected cells. (B) Immunocytochemistry analysis of H3K4me2 in GFP-Mad2l2 transfected NIH3T3 cells. Overexpression of Mad2l2 does not influence the level of H3K4me2.
(TIF)

Table S1 Mad2l2 deficient individuals appear in sub-Mendelian ratio. Numbers of animals per each genotype during embryogenesis (E8.0-E9.5 and E13.5) or after the birth are shown in percentage.
(DOCX)

Table S2 Development of ovarian structures in knockout females. 12 knockout females of different age were analyzed. In 7 animals, ovaries were not generated at all. Among the rest, 2 and 3 animals developed two or one ovaries, respectively, which lack germ cells or follicular cells (Figure 1B).
(DOCX)

Text S1 Extended Material and Methods.
(DOCX)
