# Supplementary figures and images for: A Critical Function of Mad2l2 in Primordial Germ Cell Development of Mice
Source: PLoS Genet. 2013 Aug 29;9(8):e1003712. doi: 10.1371/journal.pgen.1003712 (PMC3757036; doi:10.1371/journal.pgen.1003712)

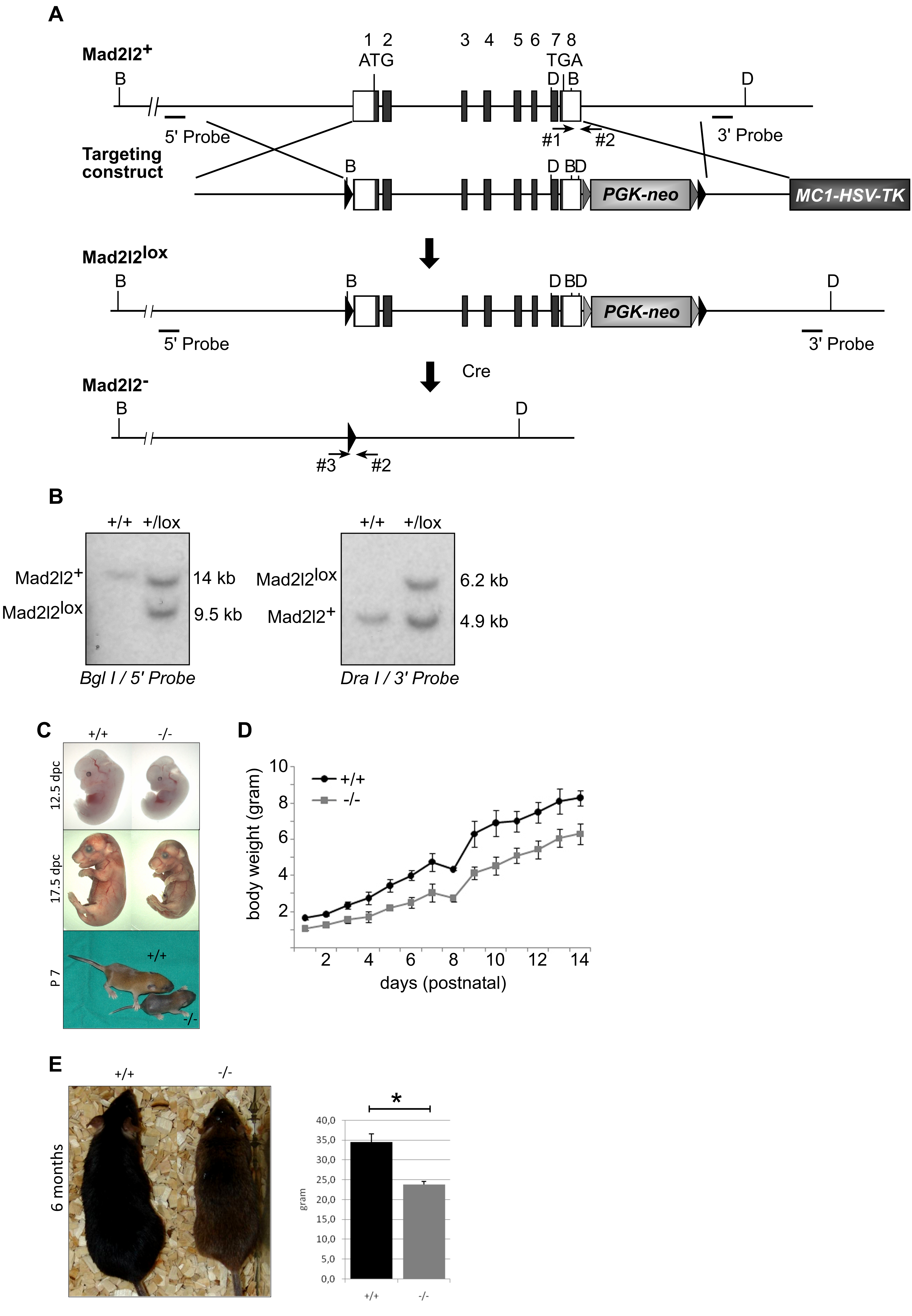

Supplement: Figure S1 — Generation and general characterization of Mad2l2 knockout mouse line. (A) Gene targeting strategy. B = Bgl1, D = Dral recognition sites. Arrows #1, 2, 3 indicate genotyping primers. (B) Confirmation of homologous recombination in Mad2l2 locus by Southern blotting of ES cells DNA. (C) Size reduction of Mad2l2 mutants. E12.5, E17.5 embryos and newborn mice on postnatal day 7 (P7) are shown. (D) Postnatal development of Mad2l2−/− mutants remains retarded. (E) Comparison of adult animals' weight shows a significant reduction in knockouts. Right graph: the average weight represented as mean ± SD of at lease three animals per each genotype. Asterisk indicates P≤0.01. (TIFF) [file pgen.1003712.s001.tiff]

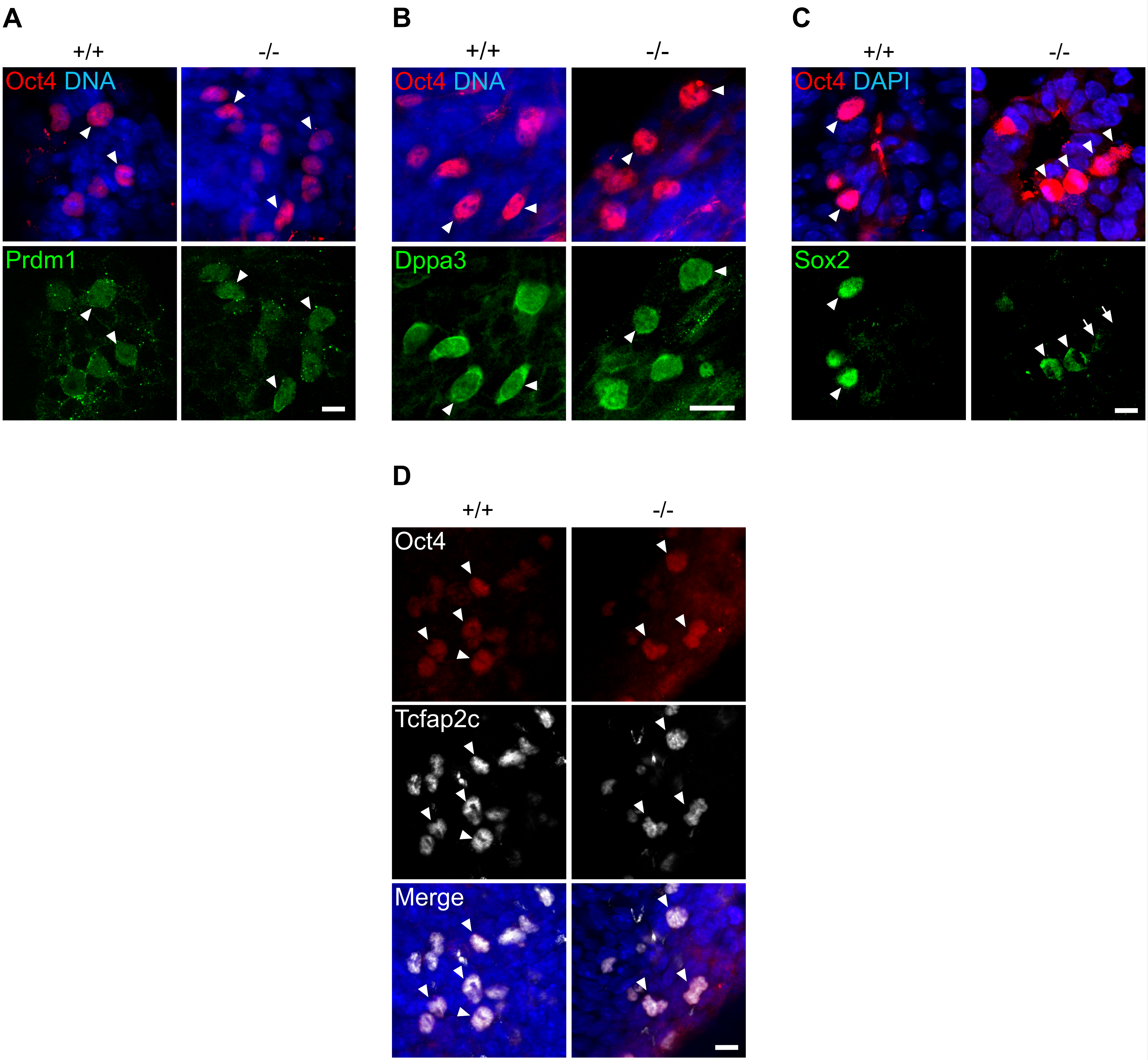

Supplement: Figure S2 — Expression of PGC-specific markers. (A,B,D) Both wild type and knockout PGCs express Prdm1, Dppa3, and Tcfap2c at E8.5. At least 50 PGCs per each genotype were analyzed. Scale bars: 20 µm. (C) Sox2 expression characterizes all Mad2l2+/+ PGCs at E9.0 (100%, 17/17). Many Mad2l2−/− PGCs of the same stage were negative for Sox2 (44%, 8/18; arrows; P≤0.05), or were only weakly positive (arrowheads). (TIFF) [file pgen.1003712.s002.tiff]

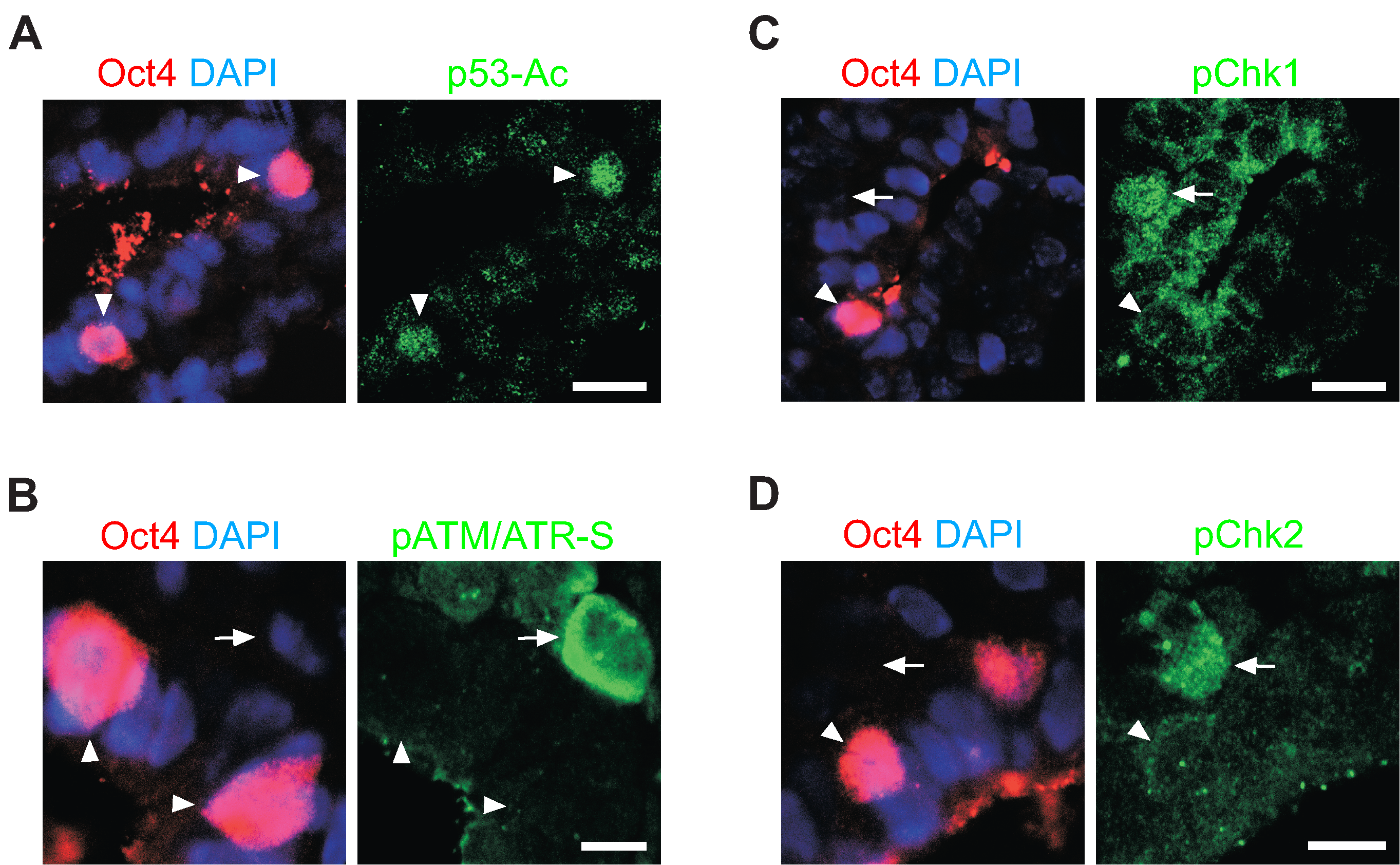

Supplement: Figure S3 — No activation of DNA damage response was observed in apoptotic Mad2l2−/− PGCs. (A) Mad2l2−/− PGCs expressed active, acetylated p53 (arrowheads, 100%, 6/6). PGCs were identified by Oct4 immunohistochemistry on transverse sections of E9.0 embryos (arrowheads). (B) No Oct4- and phospho ATM/ATR substrate-double positive PGCs were detected in Mad2l2−/− embryo section at E9.0 (arrowheads). Arrow indicates a positive somatic cell implying the proper staining. (C, D) No Oct4- and phospho-Chk1 (C) or phospho-Chk2 (D) double positive Mad2l2−/− PGCs were detected at E9.0 (arrowheads). In contrast, occasionally, some somatic cells showed expression of these active DNA damage response markers (arrows). Scale bars: A and C, 20 µm, B and D, 10 µm. (TIFF) [file pgen.1003712.s003.tiff]

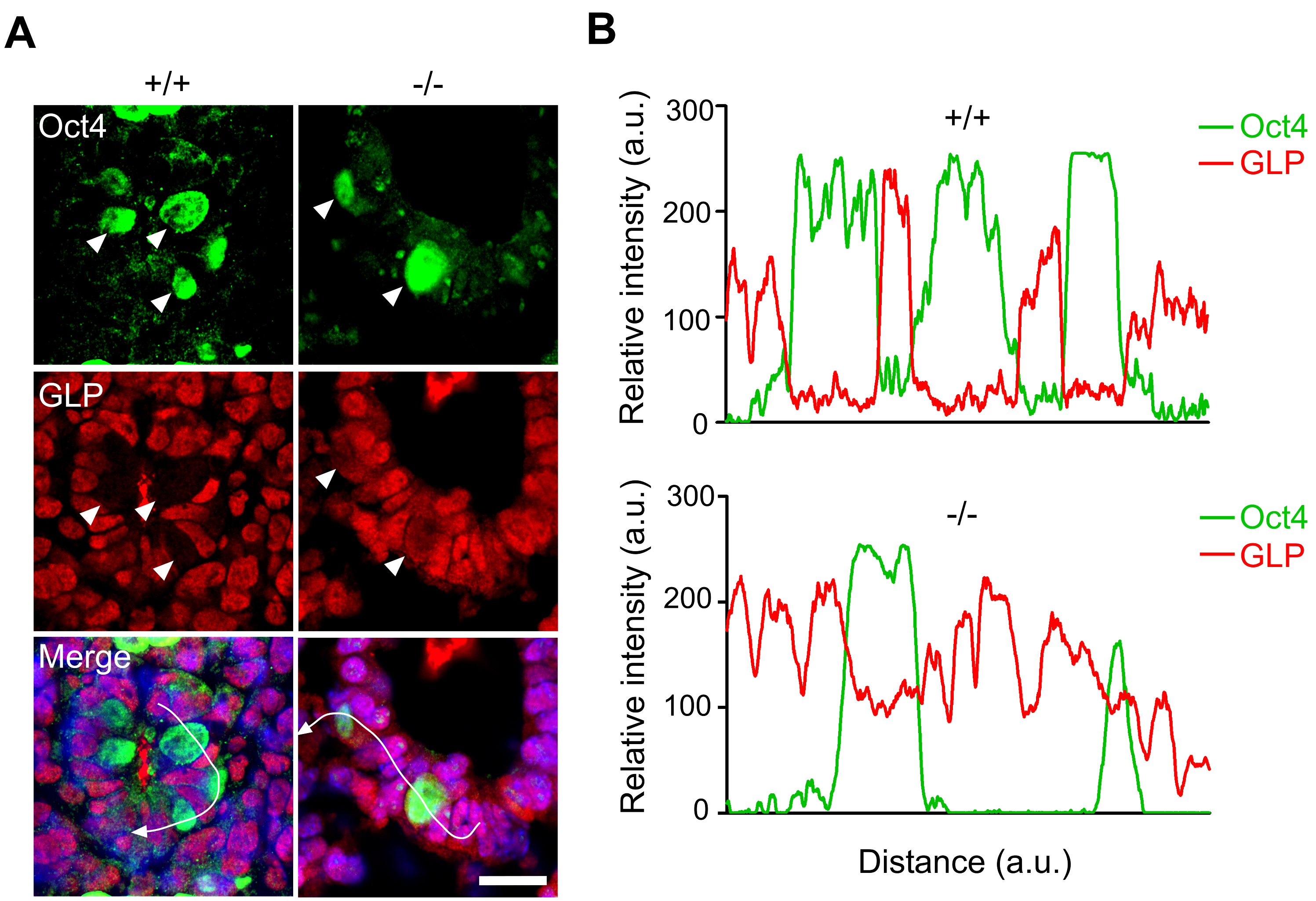

Supplement: Figure S4 — Mad2l2 deficient PGCs fail to downregulate GLP. (A) GLP expression was absent from all Mad2l2+/+ PGCs at E9.0 (arrowheads, 0%, 0/18). Most Mad2l2−/− PGCs were positive for GLP (arrowheads, 87.5%, 14/16; P≤0.05). (B) Line-scan profile of relative intensity of GLP and Oct4 fluorescent signals in (A). (TIF) [file pgen.1003712.s004.tif]

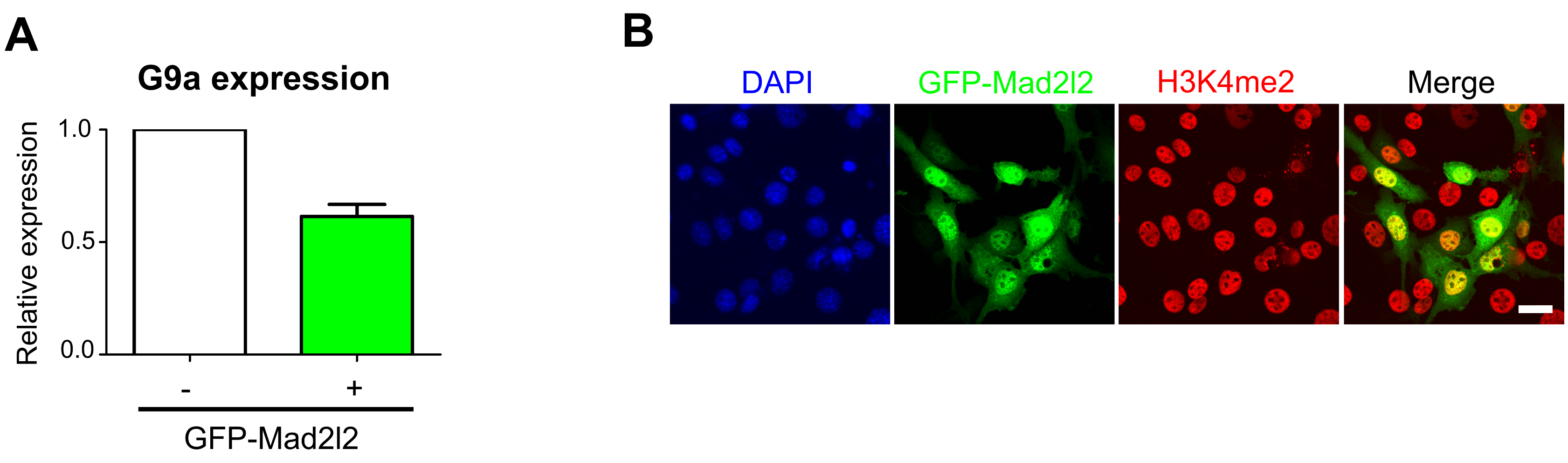

Supplement: Figure S5 — Analysis of Mad2l2 function in fibroblasts. (A) qRT-PCR analysis of G9a expression in FACS sorted NIH3T3 cells. GFP-Mad2l2 overexpression downregulates the G9a level to around half the value in non-transfected cells. (B) Immunocytochemistry analysis of H3K4me2 in GFP-Mad2l2 transfected NIH3T3 cells. Overexpression of Mad2l2 does not influence the level of H3K4me2. (TIF) [file pgen.1003712.s005.tif]
