# Supplementary material for: A Critical Function of Mad2l2 in Primordial Germ Cell Development of Mice
Source: PLoS Genet. 2013 Aug 29;9(8):e1003712. doi: 10.1371/journal.pgen.1003712 (PMC3757036; doi:10.1371/journal.pgen.1003712)
Supplement: Table S1 — Mad2l2 deficient individuals appear in sub-Mendelian ratio. Numbers of animals per each genotype during embryogenesis (E8.0-E9.5 and E13.5) or after the birth are shown in percentage. (DOCX) [file pgen.1003712.s006.docx]

Table S1

|  |  | **Number per genotype** | | | |
| --- | --- | --- | --- | --- | --- |
| **Stage** | **Litter** | **Wild type** | **Heterozygous** | **Knockout** | **Total** |
| **E8.0-E9.5** | 25 | 58 (24.78%) | 123 (52.56%) | 53 (22.64%) | 234 |
| **E13.5** | 14 | 31 (26.49%) | 66 (56.41%) | 20 (17.09%) | 117 |
| **New born** | 15 | 29 (29.29%) | 57 (57.57%) | 13 (13.3%) | 99 |
