# Supplementary material for: A Critical Function of Mad2l2 in Primordial Germ Cell Development of Mice
Source: PLoS Genet. 2013 Aug 29;9(8):e1003712. doi: 10.1371/journal.pgen.1003712 (PMC3757036; doi:10.1371/journal.pgen.1003712)
Supplement: Table S2 — Development of ovarian structures in knockout females. 12 knockout females of different age were analyzed. In 7 animals, ovaries were not generated at all. Among the rest, 2 and 3 animals developed two or one ovaries, respectively, which lack germ cells or follicular cells (Figure 1B). (DOCX) [file pgen.1003712.s007.docx]

Table S2

|  | **Age (weeks)** | **Ovarian structure** |
| --- | --- | --- |
| Female #1 | 12 | Not generated |
| Female #2 | 12 | Not generated |
| Female #3 | 11 | 1 small ovary without follicles |
| Female #4 | 13 | Not generated |
| Female #5 | 13 | 2 small ovaries without follicles |
| Female #6 | 29 | 1 small ovary without follicles |
| Female #7 | 7 | Not generated |
| Female #8 | 8 | Not generated |
| Female #9 | 22 | 2 small ovaries without follicles |
| Female #10 | 22 | Not generated |
| Female #11 | 7 | 1 small ovary without follicles |
| Female #12 | 7 | Not generated |
