# Supplementary material for: A Critical Function of Mad2l2 in Primordial Germ Cell Development of Mice
Source: PLoS Genet. 2013 Aug 29;9(8):e1003712. doi: 10.1371/journal.pgen.1003712 (PMC3757036; doi:10.1371/journal.pgen.1003712)
Supplement: Text S1 — Extended Material and Methods. (DOCX) [file pgen.1003712.s008.docx]

**Text S1. Extended material and methods.**

**Cell culture**

MEFs were prepared from individual, genotyped E13.5 embryos, and cultured as passage 0 in Dulbecco’ modified medium, 10% fetal bovine serum, penicillin/streptomycin, and sodium pyruvate (Gibco). Cells not older than passage 1 were seeded on 0.1% gelatin-coated chamber slides (Nunc). NIH 3T3 fibroblasts were cultured in the same medium, and were transfected by Lipofectamine (Invitrogen) with an expression vector for N-terminally tagged HA-Mad2l2 or GFP-Mad2l2, based on pCMV-HA (Clontech).

**Northern blot**

The northern blot membrane (Ambion) was hybridized according to instructions of the manufacturer. For an actin loading control of this northern blot see ([Pitulescu et al., 2009](#_ENREF_44)).

**Immunocytochemistry**

Cells on chamber slides were washed twice, fixed for 20 min with formaldehyde, permeablized with 0.1% Tween 20, and blocked for 1 hour in 10% normal goat serum/1% bovine serum albumin. Primary antibodies were prepared in blocking solution and applied for 1-2h at room temperature. Alexa Fluor 594 goat anti-mouse IgG, Alexa Fluor 488 goat anti-mouse IgM, Alexa Fluor 488 goat anti-rabbit IgG, Alexa Fluor 594 goat anti-rabbit IgG, Alexa Fluor 594 goat anti-rat IgG, or Alexa Fluor 488 goat anti-rat IgG were used as secondary antibodies (all from Molecular Probes). The nucleus was counterstained with 4,6-Diamidin-2-phenylindol (DAPI, Vectashield).

**Immunohistochemistry (IHC)**

Embryos were washed, fixed for 1 hour at 4° C in paraformaldehyde (PFA), washed three times, treated with 30% sucrose, immersed in a 1:1 mixture of Tissue Freezing Medium (Jung) and 30% sucrose for 30-60 min, and then embedded. 10 µm cryosections were washed, and permeablized for 10 min in 0.1% PBSTx (phosphate buffered saline/0.1% Triton X-100). Blocking was performed for 1 hour with 10% normal goat serum/1% BSA. The incubation with the primary antibody was always performed overnight at 4° C. Secondary antibodies were used as above, or the ABC staining system (Santa Cruz) was applied. For IHC analysis of global DNA methylation, E9.0 embryo sections were first stained with rabbit anti-Oct4 (Abcam), appropriate secondary antibody was applied, and then tissues were fixed with PFA, incubated with 2N HCl, permeablized and finally incubated overnight with mouse anti-5mC (Abcam) at 4° C. Related secondary antibody was used and at the end, slides were mounted and analyzed by confocal microscopy.

**Whole mount staining**

Embryos were dissected in PBS and were fixed by PFA 4% on ice for 30 minute to 1 hour, depending on the size of the embryo. PGC-containing portions of the embryos were further cut into smaller pieces to ease antibody penetration. Embryo pieces were permeablized by 1% PBSTx for 45 minutes on ice and then were incubated for 4 days with primary antibodies diluted in blocking solution (BSA 1%, normal goat serum 10% in PBSTx 1%). After washing, embryo pieces were incubated for two days with secondary antibodies and Hoechst 33258 in blocking solution then washed and mounted on slides and were finally studied by confocal microscopy (Leica, SP5). To quantify the number of PGCs, the posterior portion of the embryos was subjected to alkaline phosphatase (AP) staining by incubation in NBT/BCIP substrate solution for 4-5 min at room temperature according to the manufacturer (Roche).

**Real-Time qRT-PCR**

Total RNA from GFP-Mad2l2 FACS-sorted samples was extracted with RNeasy kit (Qiagen), and DNA was digested by DNaseI (Qiagen). cDNA was synthesized from 1 μg RNA by reverse transcriptase (Omniscript, Qiagen) and a combination of random hexamere and oligo dT primers (Promega). 25 ng cDNA per reaction was amplified by KAPA SYBR^®^ FAST qPCR Master Mix (KAPA biosystems) in Real-time PCR with an Applied Biosystems 7300 Sequence Detection system. The Ct values were determined using default threshold settings. The expression levels of samples were normalized to GAPDH.

**Apoptosis**

Programmed cell death was analyzed using the TUNEL assay (Millipore) on chamber slides or embryo cryosections. The assay was followed by immunostaining against SSEA1.

**FACS sorting**

Two days after transfection of NIH3T3 cells with GFP-Mad2l2 expressing vector, they were trypsinized, washed with PBS, and then sorted by FACS Aria II (BD). Sorted cells were lysed in RIPA buffer (for protein isolation) or in RLT buffer (for RNA isolation).

**Immunoprecipitation**

Whole cell extracts from HA-Mad2l2 transfected NIH 3T3 cells were pre-cleared with normal control IgGs (Upstate), and incubated for 1 hour at 4° C with primary antibodies. Precipitation was performed using Protein A/G PLUS-Agarose Immunoprecipitation Reagent according to manufacturer (Santa Cruz).

**Western blotting**

Protein extracts or immunoprecipitates were dissolved on gels by SDS-PAGE and then transferred to the nitrocellulose membranes. Unspecific antigens were masked by 1 hour incubation with blocking solution (5% low-fat milk dissolved in 0.1% PBSTw). Membranes were incubated with primary antibodies diluted in blocking solution overnight at 4° C on shaking plate. After three times washing with 0.1% PBSTw, membranes were incubated for 1 hour at room temperature with HRP-conjugated secondary antibodies diluted in blocking solution. Membranes were washed and developed after treating with Pico chemiluminescent substrate (Thermo Scientific).
